# “Sound” Decisions: The Combined Role of Ambient Noise and Cognitive Regulation on the Neurophysiology of Food Cravings

**DOI:** 10.3389/fnins.2022.827021

**Published:** 2022-02-16

**Authors:** Danni Peng-Li, Patricia Alves Da Mota, Camile Maria Costa Correa, Raymond C. K. Chan, Derek Victor Byrne, Qian Janice Wang

**Affiliations:** ^1^Food Quality Perception and Society Team, iSENSE Lab, Department of Food Science, Aarhus University, Aarhus, Denmark; ^2^Sino-Danish College (SDC), University of Chinese Academy of Sciences, Beijing, China; ^3^Neuropsychology and Applied Cognitive Neuroscience Laboratory, CAS Key Laboratory of Mental Health, Institute of Psychology, Chinese Academy of Sciences, Beijing, China; ^4^Department of Clinical Medicine, Center for Music in the Brain, Aarhus University, Aarhus, Denmark; ^5^Department of Psychology, University of Chinese Academy of Sciences, Beijing, China

**Keywords:** EEG, EDA, cognitive load, emotions, self-regulation, restaurant noise, decision-making, consumer behavior

## Abstract

Our ability to evaluate long-term goals over immediate rewards is manifested in the brain’s decision circuit. Simplistically, it can be divided into a fast, impulsive, reward “system 1” and a slow, deliberate, control “system 2.” In a noisy eating environment, our cognitive resources may get depleted, potentially leading to cognitive overload, emotional arousal, and consequently more rash decisions, such as unhealthy food choices. Here, we investigated the combined impact of cognitive regulation and ambient noise on food cravings through neurophysiological activity. Thirty-seven participants were recruited for an adapted version of the Regulation of Craving (ROC) task. All participants underwent two sessions of the ROC task; once with soft ambient restaurant noise (∼50 dB) and once with loud ambient restaurant noise (∼70 dB), while data from electroencephalography (EEG), electrodermal activity (EDA), and self-reported craving were collected for all palatable food images presented in the task. The results indicated that thinking about future (“later”) consequences vs. immediate (“now”) sensations associated with the food decreased cravings, which were mediated by frontal EEG alpha power. Likewise, “later” trials also increased frontal alpha asymmetry (FAA) —an index for emotional motivation. Furthermore, loud (vs. soft) noise increased alpha, beta, and theta activity, but for theta activity, this was solely occurring during “later” trials. Similarly, EDA signal peak probability was also higher during loud noise. Collectively, our findings suggest that the presence of loud ambient noise in conjunction with prospective thinking can lead to the highest emotional arousal and cognitive load as measured by EDA and EEG, respectively, both of which are important in regulating cravings and decisions. Thus, exploring the combined effects of interoceptive regulation and exteroceptive cues on food-related decision-making could be methodologically advantageous in consumer neuroscience and entail theoretical, commercial, and managerial implications.

## Introduction

### Value-Based Decision-Making

Our ability to evaluate long-term goals over immediate rewards is encoded in an array of complex computational processes in the brain (Rangel et al., 2008; Levin et al., 2012). These include resisting the impulse of consuming palatable foods, foreseeing the future potential health consequences associated, and at the same time being able to delay one’s gratification by valuing the “rational” alternative despite temporal discounting (Volkow and Baler, 2015; Cai et al., 2019).

Indeed, our choices and decisions ought to fulfill both immediate needs and those that are better served for future gains (Motoki et al., 2019). To evolutionarily optimize such balanced utilitarian behaviors, the neural circuitry of human decision-making can simplistically be divided into two neuroanatomically and -functionally distinctive systems—an automatic, emotional, impulsive system (bottom-up) and a deliberate, reflective, control system (top-down)—popularly referred to as a fast “system 1” and a slow “system 2” (Evans, 2007; Chen et al., 2018). While the emotional and motivational behaviors of system 1 are manifested in deeper striatal brain structures, the prefrontal cortices govern the cognitive and prospective system 2 functions (Peng-Li et al., 2020c).

Without cognitive inhibition of system 2, the mere presence of appetitive and salient food cues reinforces anticipatory reward (“wanting”) responses through sensitized neural firing of dopamine, potentially leading to excess food consumption, weight gain, and even addictive behaviors (Burger and Stice, 2012; Schulte et al., 2016; Coccurello and Maccarrone, 2018; Nguyen et al., 2021).

### Top-Down Cognitive Regulation

In fact, several cognitive strategies have been proposed to facilitate top-down self-regulatory eating behaviors, such as mental imagery (Petit et al., 2017; Zorjan et al., 2020) or episodic future thinking (Dassen et al., 2016; Sun and Kober, 2020). These self-managerial strategies are important components in cognitive-behavioral treatments for treating obesity, eating disorders and addictions (Grilo et al., 2011; Gearhardt et al., 2012) and have been instrumentalized in experimental paradigms (Sun and Kober, 2020).

The Regulation of Craving (ROC) task, originally developed by Kober et al. (2010a) attempts to measure the specific causal effect of regulation strategies on craving for cigarette, alcohol, and/or foods (Kober et al., 2010b; Boswell et al., 2018; Suzuki et al., 2020). The ROC task enables quantification and casual inferences of the underlying neural mechanisms of cue-induced cravings from an immediate “now” perspective (anticipatory reward) and a future “later” decision perspective (delayed gratification). For instance, using functional Magnetic Resonance Imaging (fMRI), Kober et al. (2010b) demonstrated that cravings for both cigarettes and food decreased when thinking about long-term consequences vs. immediate sensations. These subjective ratings were reflected in the blood-oxygen-level-dependent (BOLD) signal which showed that later (vs. now) -trials increased activation in the dorsomedial prefrontal cortex (dmPFC), dorsolateral prefrontal cortex (dlPFC), and ventrolateral prefrontal cortex (vlPFC)—all a part of the reflective system 2—whereas they decreased activity in brain regions associated with emotion and reward valuation (system 1), i.e., ventral striatum and amygdala.

Similarly, an electroencephalogram (EEG) study focusing on event-related potentials (ERPs), showed that a later (vs. now) mindset reduced cravings for high-caloric foods as well as evoked larger late positive potential (LPP) compared to remaining conditions, suggesting that a cognitive focus on negative long-term consequences increases arousal (Meule et al., 2013).

### Bottom-Up Auditory Manipulation

In commercial contexts, consumer researchers and behavioral economists have explored more bottom-up avenues for alleviating the “obesogenic” environment. Such sensory marketing strategies entail changing the so-called choice architecture by nudging consumers toward healthier behaviors through multisensory cues in the environment (Krishna, 2012; Bucher et al., 2016; Seo, 2020). Particularly, auditory contributions to this field have in the past decade emerged with numerous studies highlighting the often underestimated power of sound and noise on food choice (Huang and Labroo, 2019), liking (Alamir and Hansen, 2021), attention (Peng-Li et al., 2020b), and perception (Woods et al., 2011).

Louder (vs. softer) ambient noise has consistently shown adverse effects on psychophysiological mechanisms, including increased arousal states (Alvarsson et al., 2010) and cognitive load (Mehta et al., 2012), potentially resulting in poorer decisions and unhealthier food choices (Biswas et al., 2019; Volz et al., 2021; Peng-Li et al., 2022). These phenomena can be explained through the lenses of attentional processes and sensory overload (Doucé and Adams, 2020), whereby “*louder noise may diminish the ability to attend to specific elements of the experience*” (Bravo-Moncayo et al., 2020). In fact, attentional distractions have been associated with decreased functional brain connectivity between the inferior frontal gyrus (part of system 2) and the putamen (part of system 1) during goal-directed effort for food rewards (Duif et al., 2020). A complementary mechanism can be reasoned through evidence of sensation transference (Spence and Gallace, 2011), affective priming (Tay and Ng, 2019), or embodied cognition (Zhu and Meyers-Levy, 2005), all in which the ambient sounds physiologically change consumers’ interoceptive, reward, and emotional responses (Salimpoor et al., 2011; Liu et al., 2018; Kantono et al., 2019).

### Conceptual Framework

The evidence highlighted thus far conveys that our food cravings are driven by how we internally are able to regulate our valuation and decisions processes (system 1 or system 2), but at the same time, sensory distractions, such as ambient noise, are also influencing our cognitive resources and emotional states necessary for controlling and managing these behaviors. This implies that the underlying mechanisms of food-related decision-making are based on an integration of exteroceptive sensory inputs and interoceptive bodily states (Petit et al., 2016; Papies et al., 2020), that translate our somatic signals into feelings of anticipation, desires, or cravings (Bechara et al., 2005; He et al., 2019).

To understand these different, yet possibly interacting factors, on a behavioral as well as neural level, the employment of implicit psychophysiological measures can be advantageous. One approach to assess this is through EEG. In addition to the measurement of electrophysiological activity response to a specific single time-locked stimulus or event as in ERP research (Shang et al., 2018), longer-lasting and continuous functional indices of neural activity are also possible via EEG (Fernandez Rojas et al., 2020; Firestone et al., 2020; Diao et al., 2021). Here, the EEG signal can be decomposed into various frequency spectra representing the oscillatory dynamics in the brain and correlated with certain mental processes (Barlaam et al., 2011; Diao et al., 2017; Aoh et al., 2019). In fact, the power spectral density (PSD) in specific frequency bands, e.g., theta (4–8), alpha (8–12 Hz), and beta (12–25 Hz), have been associated with various distinct cognitive and emotional states during food viewing (Tashiro et al., 2019; Biehl et al., 2020) and music/noise listening (Gleiss and Kayser, 2014; Chabin et al., 2020).

In the decision and cognitive science literature, both theta and alpha activity in frontal and parietal regions are commonly linked to measures of cognitive load, i.e., the used amount of working memory recourses (Stipacek et al., 2003; Antonenko et al., 2010; Brouwer et al., 2012), including focused attention and sensory processing (Cabañero et al., 2019). Particularly, spectral theta power has been found to increase with sustained concentration and task difficulty (Gevins and Smith, 2003), while alpha oscillatory activity has been associated with alertness (Kamzanova et al., 2014) and cognitive fatigue (Borghini et al., 2012). Likewise, a large body of evidence suggests that augmented PSD in the beta frequency band is related to active and analytical thinking (Zhang et al., 2008) as well as short-term memory (Palva et al., 2011) and mental workload (Coelli et al., 2015). Of course, delta and gamma band power have also been explored in the context of human behavior (Posada-Quintero et al., 2019), yet they are less related to cognitive and mental workload in decision research (Fernandez Rojas et al., 2020).

Instead, frontal lateralization, commonly referred to as frontal asymmetry (FA; Ramsøy et al., 2018), especially in the alpha frequency range, FA has been employed as an index of mental engagement, reward anticipation, and incentive salience and shown to converge with BOLD activity in frontal cortices (Gorka et al., 2015). In particular, greater right (vs. left) frontal hemispheric alpha power is indexed by a positive frontal alpha asymmetry (FAA) score, denoting emotional motivation and approach, whereas a negative FAA score is linked to avoidance and withdrawal behavior (van Bochove et al., 2016; Fischer et al., 2018). Preliminary evidence even suggests that FAA functions as a potential biomarker for affective neuromodulation (Sun et al., 2017). FAA might therefore be a useful measure for studying affective states and cognitive processes in response to multisensory stimuli.

In short, EEG frequency patterns can be an excellent tool and for measuring the underlying brain dynamics of food-related and managerial decision-making processes. Through spectral analyses, it offers an implicit, objective, and nuanced quantification of cognitive load and related emotional processes, which is not restrained by introspection, verbalization, or any other subjective and self-report limitations.

Similarly, measurements based on the sympathetic activity in the peripheral nervous system, including electrodermal activity (EDA), also referred to as galvanic skin response (GSR) can generate complementary biometric information of these affective processes. That is, EDA amplitude amplification, thereby higher EDA peak probability has been used to capture increased emotional arousal states. With increased sympathetic activity due to interoceptive or exteroceptive triggers, sweat production is elevated, leading to heightened/lowered skin conductance/resistance as an indication of elevated arousal (Kytö et al., 2019; Verastegui-Tena et al., 2019; Pedersen et al., 2021), as determined by the circumplex model of affect (Russell, 1980).

**FIGURE 1:**
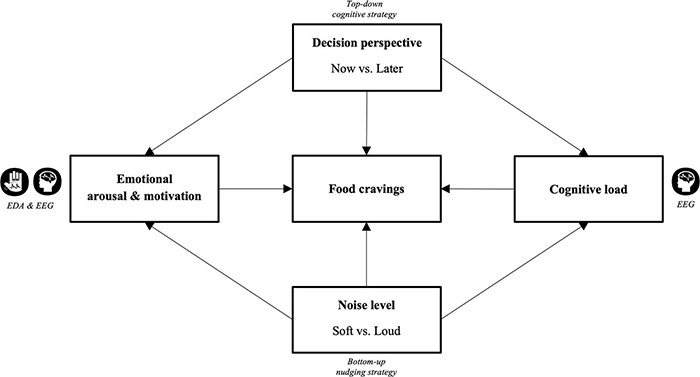
Conceptual framework. Exploring the effects of top-down cognitive strategy (now vs. later decision perspective) and bottom-up nudging strategy (soft vs. loud ambient restaurant noise) on food cravings by means of cognitive load (EEG), emotional motivation (EEG), and emotional arousal (EDA).

In light of the empirical framework, we here investigated the influences of self-regulatory decision strategy and ambient noise level on cue-induced food cravings by means of neurophysiological activity. We adapted an EEG-based ROC task (Kober et al., 2010b; Meule et al., 2013) in which participants should either focus on the long-term consequences or the immediate rewards of eating high-caloric palatable foods while listening to either soft or loud levels of restaurant noise. We hypothesized that both noise level and decision perspective would affect subjective food cravings as well as objective measures, including EDA and EEG, as measures of emotional arousal/motivation and cognitive load (Figure 1). Specifically, we expected, as a result of increased emotional arousal and motivation as well as cognitive load, that loud noise would potentially diminish the cognitive resources requisite for more top-down processing, important for especially thinking about future consequences associated with the food. To test this, we examined the PSD in the theta, alpha, and beta frequency bands in the fronto-cortical areas, FAA, as well as EDA during cognitive regulation in the presence of ambient noise and visual food presentation.

## Materials and Methods

### Participants

Thirty-seven healthy Danish university students aged 18–35 years were recruited through the Sona recruitment system at the Cognition and Behavior (COBE) Lab, Aarhus University, Denmark.^1^ The choice of sample size was based on previous EEG literature employing similar designs (*n* = 25; Meule et al., 2013; *n* = 28; Biehl et al., 2020; *n* = 19; Tashiro et al., 2019). As this is the first study implementing these conditions/manipulations, we computed a hypothetical power calculation in G*power (Faul et al., 2009). This yielded a required sample size of at least 28 participants at a power of 0.95, effect size of 0.1, and α of 0.05. All participants fulfilled the screening criteria and reported having a normal or corrected-to-normal hearing, normal or corrected-to-normal vision without color blindness, no food allergies, no dietary restraints, and no cardiovascular or neurological diseases. One participant was omitted from the analysis due to unacceptable data quality, resulting in a valid sample size of 36 (mean age ± SD = 24.22 ± 3.59 years; mean BMI ± SD = 23.52 ± 3.90 kg/m^2^; 50% females) all of whom provided written informed consent. The study was approved by the Aarhus University Ethics Committee (approval number: 2020-0184772) and conducted in accordance with the ethical standards laid out in the Declaration of Helsinki. All participants were compensated monetarily for their participation (250 DKK).

### Regulation of Craving Task

**FIGURE 2:**
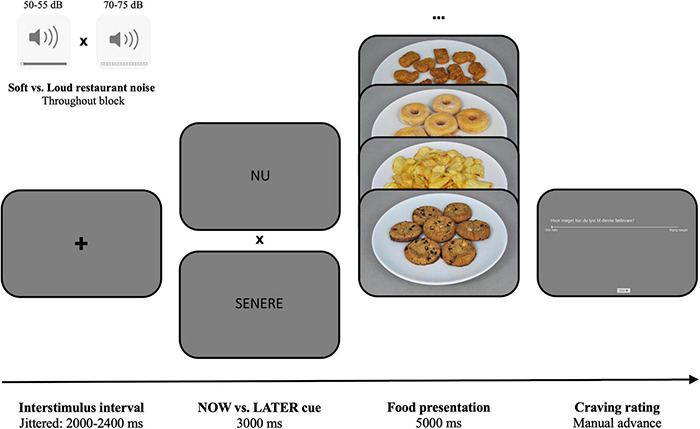
The adapted ROC task. Before each trial, a jittered inter-trial interval (fixation cross) is presented for 2–2.4 s. Then either a now or later cue (nu or senere in Danish) is shown for 3 s, followed by 5 s exposure of a high-calorie food item. Finally, participants rate how much they want the presented food on a VAS from “not at all” to “very much.” Either soft or loud noise is played in the background throughout the entire block.

The ROC task experimentally measures the specific causal effect of regulation strategies and self-management on craving, as well as allows to study its underlying neural mechanisms. The original ROC used images of cigarettes and unhealthy foods to induce cravings among cigarette smokers (Kober et al., 2010a). In our adapted version, we exclusively focused on high-calorie food items as craving cues. During each trial of the adapted ROC task (Figure 2), participants were exposed to one of these cues, preceded by the instruction to follow one of two decision perspectives: “now”—focus on the immediate sensations and feelings associated with consuming the food (e.g., it will taste good and satisfy my cravings), or “later”—focus on the long-term negative consequences associated with repeated consumption (e.g., it will increase my risk for weight gain and heart disease). Participants were then asked to rate their craving for the specific food they just saw (“how much do you crave this food?”), using a 1 (not at all) to 5 (very much) visual analog scale (VAS). The now or later instructions were presented for 3,000 ms and the subsequent food image for 5,000 ms. Between each trial, a jittered 2,000–2,400 ms fixation cross was inserted. We implemented 60 different trials (30 now-trials and 30 later-trials) per experimental block, which was repeated for each of the two sound conditions (soft noise vs. loud noise), resulting in a total of 120 trials in the experiment. Trials were presented in a randomized order and blocks were counterbalanced across participants. The adapted ROC task was programmed in the iMotions software (Copenhagen, Denmark)^2^.

### Self-Regulation of Eating Behavior Questionnaire

The 5-item Self-Regulation of Eating Behavior Questionnaire (SREBQ) is a measure of eating self-regulatory capacity (Kliemann et al., 2016). The SREBQ assesses people’s capacity to control and manage their eating behavior in order to achieve and/or maintain their eating intentions. We adapted the original SREBQ into a Danish version using back-translation. The total score cut-off points include < 2.8 = low self-regulation, 2.8–3.6 = medium self-regulation > 3.6 = high self-regulation.

### Visual Stimuli

Thirty high-resolution standardized high-caloric food images from the *Full4Health Image Collection* (Charbonnier et al., 2016) were selected for the current study (mean_calorie_ = 384 kcal/100 g; mean_fat_ = 21 g/100 g). The images were balanced in terms of taste, such that 15 images were categorized as sweet food items and 15 as savory food items (Table 1). The images were taken in a closed 60 × 60 × 60 cm cubic photo tent. Two daylight lamps (E27/55W) were used to create optimal lighting conditions. The lens angle was approximately 45°, the distance from center plate to center tripod was 39.5 cm, and the height of the center of the camera on the tripod was 38 cm to resemble the viewing of a plate of food on a table during mealtime. Each food was presented on a white plate with a diameter of 17.0 cm. A light gray background was chosen to ensure sufficient contrast between plate and background. To standardize the background, MeVisLab (MeVis Medical Solutions AG, Bremen, Germany) and the open-source registration software Elastix^3^ were used (Klein et al., 2010). Each plate was segmented, registered on a standardized background from one image, and smoothened on the plate edges. The complete photographing protocol is described in Charbonnier et al. (2016).

**TABLE 1:** Calorie and fat content per 100 g of the 30 food images included in the study.

Food item	Taste category	Calorie (kcal/100 g)	Fat (g/100 g)	Image no.
Potato crisps (natural)	Savory	541	33.5	1
Spring rolls	Savory	181	8.2	10
Chicken nuggets	Savory	272	17.1	12
French fries	Savory	306	14.3	16
Nacho-cheese tortilla chips	Savory	487	22.3	24
Pepper potato crisps	Savory	544	33.0	122
Croissants	Savory	424	23.0	130
Wotsits cheesey (chips)	Savory	547	33.0	185
Pizza Bolognese	Savory	234	9.5	245
Paprika chips	Savory	544	33.0	316
Cheese burgers	Savory	246	12.0	317
Pita with doner	Savory	218	14.0	318
Turkish pizza with doner	Savory	233	10.0	319
Pizza margarita	Savory	251	12.3	321
French fries with ketchup	Savory	268	11.9	322
Donuts with icing	Sweet	416	27.8	25
Chocolate chip cookies	Sweet	500	25.0	26
Milk chocolate	Sweet	546	32.5	32
Chocolate nuts	Sweet	584	42.1	36
Brownies	Sweet	401	20.0	43
Whipped cream pie	Sweet	350	25.0	44
Mini donuts	Sweet	358	21.1	100
Pancakes	Sweet	196	4.9	101
Syrup waffles	Sweet	473	19.3	109
Cake with chocolate	Sweet	450	25.0	112
Strawberry pie	Sweet	205	11.0	117
Cake	Sweet	424	23.9	118
Round pastry/danish	Sweet	315	9.0	289
Knoppers	Sweet	548	33.4	302
Prince biscuits	Sweet	469	17.0	304
*Average*		*384*	*21*	

*Image no. refers to the Full4Health Image Collection numbering (Charbonnier et al., 2016).*

### Auditory Stimuli

Two versions of a restaurant noisescape (chattering and tableware noises) retrieved from Freesound^4^ were used for the study. The volume level of the noisescape was manipulated based on the Loudness Unit Full Scale (LUFS) by the European Broadcast Union (EBU) standards (European Broadcast Union, 2016). To attain a soft volume version, the noisescape was decreased to approximately –30 LUFS, while the loud version was increased to approximately –4 LUFS via Logic Pro Version 10.6.1 (Apple Inc.). This was done to ensure the sound intensity (dB) matched 50–55 dB (soft) and 70–75 dB (loud) after audio calibration. The volume levels were chosen based on prior research, which has indicated sound at 80 dB leads to negative affect and even loss of hearing, and sound below 50 dB is often not detected (Witt, 2008). Furthermore, previous food-sound studies have used sound/noise levels in similar ranges (Woods et al., 2011; Biswas et al., 2019). The two noisescapes were first validated in a separate online test (*N* = 91) in which participants listened to each version and rated them in terms of relaxation/arousal on a VAS from 1 to 9. Soft restaurant noise (mean rating ± SD = 4.27 ± 2.25) was expectably perceived as being more relaxing (vs. arousing) compared to loud restaurant noise (mean rating ± SD = 7.49 ± 1.04). The final noisescapes used for the study can be heard at: https://soundcloud.com/danni-peng-li/sets/eeg-roc-t-sound-study.

### Design and Procedure

To control for possible hunger effects, participants were asked to fast for 2 h (i.e., no food intake but water intake was allowed) and refrain from consuming alcoholic drinks for 24 h prior to the study (Frank et al., 2010; Hume et al., 2015; Zhang and Seo, 2015). On testing days (between 9 am to 5 pm), participants arrived at the laboratory for a 1.5 h session where they were informed about the study procedure and provided written informed consent. Participants were seated 70 cm from the HP EliteDisplay E243i, 24,” 16:10 monitor (screen resolution of 1,920 × 1,080 pixels), while EEG and EDA electrodes were applied while checking signal quality in the iMotions software. No natural light entered the room (i.e., only artificial LED light). To reduce movement artifacts participants rested their heads on a chinrest attached to the table. During the paradigm introduction, participants were instructed to minimize head movements throughout the recordings. They also rated how hungry they were on a 9-point VAS. They then completed 4 practice trials to familiarize themselves with the task. After ensuring that participants understood the procedure, they initiated the two counterbalanced experimental blocks (conditions) of the adapted ROC-task—one block with soft ambient restaurant noise and one block with loud ambient restaurant noise—with a 5 min break between blocks and an optional break within each block. The adapted ROC task was followed by a manipulation check, i.e., arousal, valence, and distraction ratings of the noisescapes on a 9-point VAS, as well as completion of the SREBQ. Finally, demographic information was collected.

### Signal Processing

**FIGURE 3:**
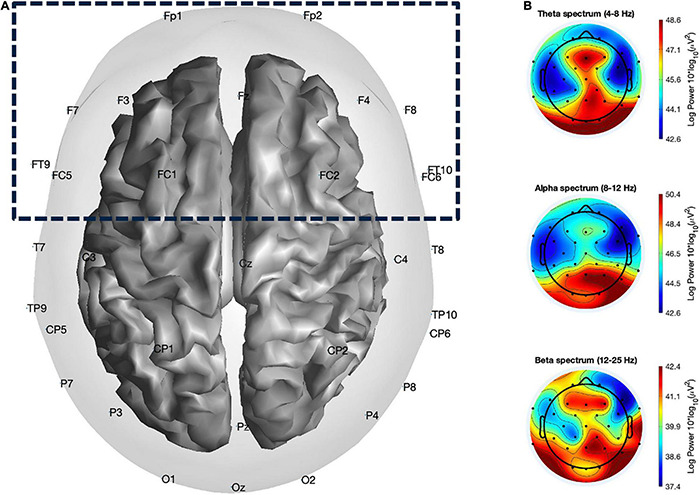
Illustration of **(A)** channel locations of the 32 electrodes with the frontal ROI highlighted, including Fp_1_, Fz, F_3_, F_4_, FT_9_, FC_5_, FC_7_, FT_10_, FC_6_, FC_2_, F_4_, F_8_, Fp_2_, and **(B)** example topographical maps of theta (4–8 Hz), alpha (8–12 Hz), and beta (12–25 Hz) power band activity across conditions.


EEG data were collected from 32 Ag/AgCl electrodes (Fp_1_, Fz, F_3_, F_4_, FT_9_, FC_5_, FC_7_, C_3_, T_7_, TP_9_, CP_5_, CP_1_, Pz, P_3_, P_7_, O_1_, Oz, O_2_, P_4_, P_9_, TP_10_, CP_6_, CP_2_, Cz, C_4_, T_8_, FT_10_, FC_6_, FC_2_, F_4_, F_8_, Fp_2_) placed according to the 10–20 system using actiCap (Brain Products GmbH, Gilching, Germany) with a sampling rate of 500 Hz. Raw EEG data were filtered (Butterworth) with a zero phase-lag band-pass filter [0.5–100 Hz] and a zero phase-lag notch filter (50 Hz), re-referenced to the mastoid reference electrode placed at TP9. Artifacts were then rejected using an artifact threshold [120 μV] based on the absolute signal value. Power spectra analysis was computed using Fast Fourier Transform (FFT; Welch method; Welch, 1967), by splitting pre-processed data into 1-s time windows with an overlap of 50% and submitted to the FFT, resulting in one power spectrum per 0.5 s. Finally, theta, alpha, and beta activities were calculated by averaging the power spectral density within the standard power bands: theta [4–8 Hz], alpha [8–12 Hz], and beta [12–25 Hz] (Figure 3B). We focused on a hypothesis-based region of interest (ROI) by clustering the frontal electrodes (Fp_1_, Fz, F_3_, F_4_, FT_9_, FC_5_, FC_7_, FT_10_, FC_6_, FC_2_, F_4_, F_8_, Fp_2_; Figure 3A). This electrode clustering was chosen based on previous literature showing various cognitive processes related to the multiple frontal regions as described in the “Introduction” section as well as to avoid loss in statistical power (Moazami-Goudarzi et al., 2008). Furthermore, FAA scores were computed using two frontal electrodes (F_3_ and F_4_) on each hemisphere using the formula according to Allen et al. (2004):

$$FrontalAlphaAsymmetry\left(FAA\right)=\text{ln}\left(\frac{\alpha_{F_{4}}}{\alpha_{F_{3}}}\right)$$

EDA data was collected from two analog electrode channels placed on the tip of the fingers using a Shimmer3 GSR+ (Shimmer Sensing, Dublin, Ireland). The phasic signal was extracted using a median filter over a time window of 8,000 ms, and a low-pass Butterworth filter with a cutoff frequency of 5 Hz was applied to the phasic signal. Peak onset thresholds [0.01 μS] and offset thresholds [0 μS] were then detected on the phasic signal. EDA peak amplitude threshold was set at 0.005 μS with a minimum peak duration of 500 ms. All physiological measures were enclosed to a time window of 5 s, i.e., during food presentation in order to capture audiovisual stimulations of food and noise. Signal processing steps for EEG and EDA were carried out in iMotions through an integrated R algorithm.

### Data Analysis

All physiological and behavioral data were imported and analyzed in R version 4.0.2 for Mac OS. A manipulation check was performed using a pairwise *t*-test based on VAS ratings to ensure that the two soundscapes were in fact perceived differently in terms of arousal (1 = very relaxing; 9 = very arousing), valence (1 = very pleasant; 9 = very unpleasant), and distraction (1 = not distracting at all; 9 = very distracting).

To investigate the effects of ambient noise and cognitive regulation strategy on EEG, EDA, and self-reported cravings, we carried out generalized linear mixed models (GLMMs) via the *glmer()*-function of the *lme4* package. The GLMMs account for the hierarchical structure, non-independence of observations from individual participants in the repeated measure design, and to satisfy the normality assumptions without transformation. EEG and craving data were fitted using a *Gaussian* distribution with the *restricted maximum likelihood* (*REML*) method (Heller et al., 2016), while EDA peak detection was fitted using *Poisson* distribution (Bolker et al., 2009). In all models, the independent variables were noise level (soft vs. loud) and decision perspective (now vs. later), which were coded as fixed effects. Participant ID entered the model as a random effect. Furthermore, we controlled for possible confounds by adding BMI, hunger status, and SREBQ scores as covariates to the models. However, none of the covariates contributed significantly to any of the models, and as we did not have any *a priori* hypotheses regarding these factors, they were therefore removed from the analyses [BMI_theta_: *F*_(1, 34)_ = 0.39; *p* = 0.538; BMI_alpha_: *F*_(1, 34)_ = 0.26; *p* = 0.614; BMI_beta_: *F*_(1, 34)_ = 0.21; *p* = 0.653; Hunger_theta_: *F*_(1, 34)_ = 1.45; *p* = 0.237; Hunger_alpha_: *F*_(1, 34)_ = 0.33; *p* = 0.567; Hunger_beta_: *F*_(1, 34)_ = 3.35; *p* = 0.076; SREBQ_*theta*_: *F*_(1, 34)_ = 0.11; *p* = 0.738; SREBQ_alpha_: *F*_(1, 34)_ = 0.53; *p* = 0.470; SREBQ_beta_: *F*_(1, 34)_ = 0.46; *p* = 0.502]. The dependent variables of interest included frontal theta power, frontal alpha power, frontal beta power, FAA, EDA peaks, and food craving. Omnibus tests were carried out to test the main effects and interactions between the fixed independent variables. If a significant interaction was indicated by the GLMM, Tukey’s HSD *post hoc* tests were performed to explore the corrected pairwise comparisons.

Finally, to theorize our conceptual model, we computed four conjoint multiple mediation analyses using the *lavaan* structural equation modeling package (Rosseel, 2012). Noise level and decision perspective, respectively, entered the models as the binary independent/exogenous variables, craving as the dependent/endogenous variable, and measures of cognitive load (frontal theta power, frontal alpha power, and frontal beta power) as well as emotional arousal (EDA) and emotional motivation (FAA) as the mediators. The multiple mediation analyses were carried out using bootstrapping procedure with the *DWLS* estimator for 1,000 bootstrapped samples.

## Results

### Manipulation Check

In terms of arousal, the loud noise (mean rating ± SD = 7.22 ± 1.37) compared to the soft noise (mean rating ± SD = 3.99 ± 1.71) was perceived as being significantly more arousing [vs. relaxing; *t*_(35)_ = 10.98; *p* < 0.001]. For valence, the soft noise (mean rating ± SD = 4.03 ± 1.60) compared to the loud noise (mean rating ± SD = 6.88 ± 1.64) was likewise perceived as being significantly more pleasant [vs. unpleasant; *t*_(35)_ = 8.53; *p* < 0.001]. Finally, with regard to distraction, the loud noise (mean rating ± SD = 7.57 ± 1.34) compared to the soft noise (mean rating ± SD = 3.73 ± 1.81) was perceived as being significantly more distracting [*t*_(35)_ = 12.86; *p* < 0.001].

### Behavioral Analysis

**FIGURE 4:**
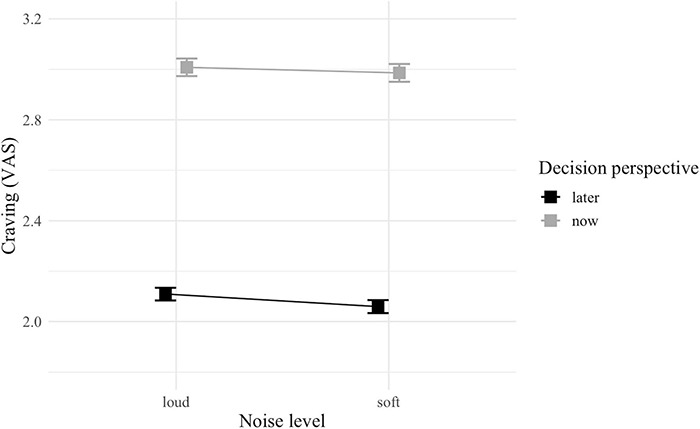
Interaction plot of self-reported cravings between noise level (soft vs. loud) and decision perspective (now vs. later). Error bars represent standard error.

The GLMM did not detect any significant interaction, but a main effect of decision perspective was observed with food cravings being reportedly significantly stronger in now (vs. later) -trials [*F*_(1, 4222)_ = 1,032.92; *p* < 0.001; Table 2 and Figure 4].

**TABLE 2:** Overview of the GLMM omnibus tests for self-reported cravings.

**Craving**			
*Fixed effects*	*F*	*df*	*p*
Noise level	2.16	1, 4230	0.141
Decision perspective	1,032.92	1, 4222	< 0.001***
Noise level × Decision perspective	0.24	1, 4222	0.623

***p < 0.001.

### Electroencephalography Power Spectral Analysis

**FIGURE 5:**
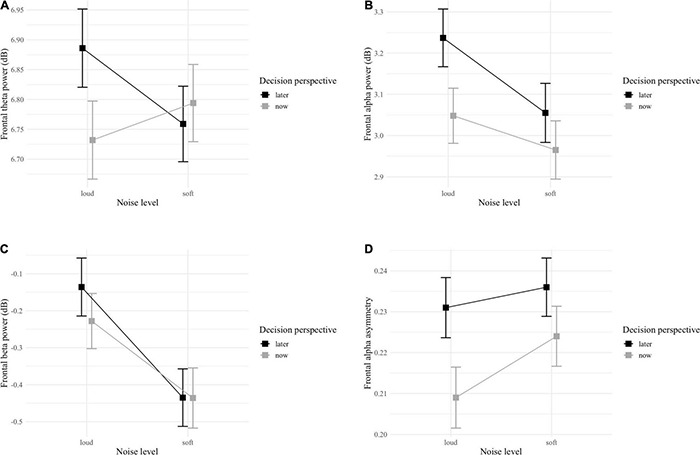
Interaction plots of **(A)** frontal theta power, **(B)** frontal alpha power, **(C)** frontal beta power frontal, and **(D)** alpha asymmetry between noise level (soft vs. loud) and decision perspective (now vs. later). Error bars represent standard error.

For frontal theta power, the GLMM indicated a significant interaction effect between noise level and decision perspective [*F*_(1, 4222)_ = 5.49; *p* = 0.019; Table 3 and Figure 5A]. *Post hoc* analyses showed that only in the loud noise condition, the theta band power was stronger for later (vs. now) decisions [*z*_(1609)_ = 2.72; *p* = 0.033]. The GLMM for frontal alpha power did not detect any significant interaction but, a main effect of both noise level and decision perspective was observed with alpha band power being stronger during the loud noise [*F*_(1, 4222)_ = 10.59; *p* = 0.001] and later decision perspective [*F*_(1, 4222)_ = 16.49; *p* < 0.001] conditions (Table 3 and Figure 5B). Similarly, the GLMM for frontal beta power did not detect any significant interaction, but a main effect of noise level was observed with beta band power being stronger in the loud noise condition [*F*_(1, 4222)_ = 12.86; *p* < 0.001; Table 3 and Figure 5C]. Finally, for FAA, the GLMM did not detect any significant interaction, but a main effect of decision perspective was observed with FAA being higher in the later decision perspective condition [*F*_(1, 4222)_ = 6.08; *p* = 0.014; Table 3 and Figure 5D].

**TABLE 3:** Overview of the GLMM omnibus tests for frontal theta power, frontal alpha power, frontal beta power, and frontal alpha asymmetry.

**EEG frontal theta power**			
*Fixed effects*	*F*	*df*	*p*
Noise level	0.31	1, 4222	0.576
Decision perspective	2.18	1, 4222	0.140
Noise level × Decision perspective	5.49	1, 4222	0.019*
**EEG frontal alpha power**			
*Fixed effects*	*F*	*df*	*p*
Noise level	10.59	1, 4222	0.001**
Decision perspective	16.49	1, 4222	<0.001***
Noise level × Decision perspective	2.05	1, 4222	0.152
**EEG frontal beta power**			
*Fixed effects*	*F*	*df*	*p*
Noise level	12.86	1, 4222	<0.001***
Decision perspective	1.35	1, 4222	0.245
Noise level × Decision perspective	1.29	1, 4222	0.257
**EEG frontal alpha asymmetry**			
*Fixed effects*	*F*	*df*	*p*
Noise level	1.92	1, 4222	0.166
Decision perspective	6.08	1, 4222	0.014*
Noise level × Decision perspective	0.55	1, 4222	0.457

*p < 0.05; **p < 0.01; ***p < 0.001.

### Biometric Analysis

**FIGURE 6:**
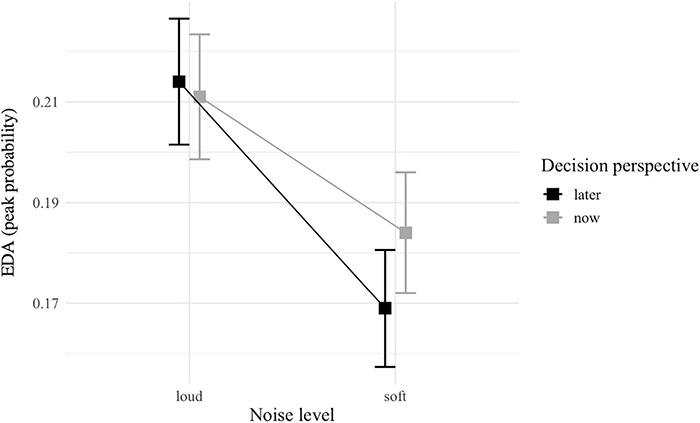
Interaction plot of EDA peaks between noise level (soft vs. loud) and decision perspective (now vs. later). Error bars represent standard error.

The EDA-based GLMM did not detect any significant interaction, but a main effect of noise level was observed with a higher probability of EDA peak threshold during loud (vs. soft) noise [*z*_(4122)_ = 3.27; *p* = 0.001; Table 4 and Figure 6].

**TABLE 4:** Overview of the GLMM omnibus tests for EDA peaks.

**EDA peaks**			
*Fixed effects*	*z*	*df*	*p*
Noise level	3.27	4122	0.001**
Decision perspective	0.15	4122	0.874
Noise level × Decision perspective	0.87	4122	0.384

**p < 0.01.

### Multiple Mediation Analysis

**FIGURE 7:**
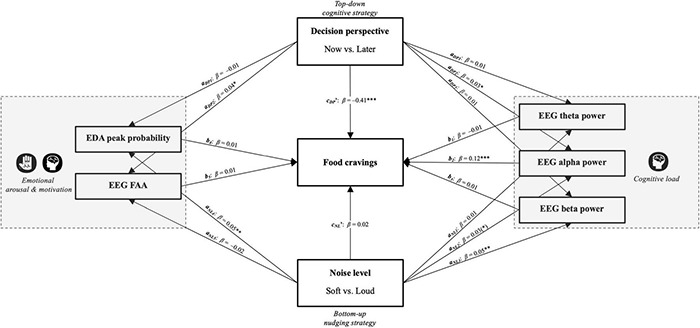
Diagram of the multiple mediation analyses based on our conceptual framework in Figure 1 with noise level (_NL_) and decision perspective (_DP_) as the independent variables, cravings as the dependent variable, and frontal theta power, frontal alpha power, frontal beta power and EDA as the mediators. Paths are shown with standardized regression coefficients and *p*-values (*p < 0.05; **p < 00.01; ***p < 0.001).

Figure 7 illustrates all of the regression coefficients between independent variables and the mediators as well as the pathways from the mediators onto the dependent variable. With noise level (_NL_) as the independent variable, the mediation analysis indicated that the standardized indirect effects of neither cognitive load measures (frontal theta power, frontal alpha power, and frontal beta power) nor emotional measures (EDA and FAA) were significant, although frontal alpha power denoted a trend (a_NL2_*b_2_; β = 0.01; *z* = 1.76; *p* = 0.079). Similarly, the direct effect of noise level on cravings was insignificant (*c_*NL*_’*; β = 0.02; *z* = –1.34; *p* = 0.179). With decision perspective (_*DP*_) as the independent variable, the mediation analysis signified that the standardized indirect effects of frontal alpha power were significant (a_DP2_*b_2_; β = 0.01; *z* = 1.95; *p* = 0.050), while the remaining mediators were not. Once this mediator was accounted for, there was still a significant direct effect of decision perspective on cravings (*c_*DP*_’*; β = –0.41; *z* = 30.69; *p < 0.*001), suggesting a partial mediation effect of the frontal EEG alpha power on self-reported food cravings.

## Discussion

While a body of psychiatric and neuroscientific research has investigated the impact of top-down cognitive strategies, self-regulation, and managerial decision-making on the neurophysiological underpinnings of food cravings, empirical findings in sensory and consumer science have shown that bottom-up auditory nudging strategies can also influence eating motivation and food valuation. In the current study, we explored both avenues in a single experimental paradigm employing an adapted version of the ROC task.

Our findings do not only provide direct support for our hypothesis that prospectively thinking about long-term consequences can effectively reduce food cravings as demonstrated in Kober et al. (2010a), but simultaneously our results suggest that the underlying causal mechanisms of these self-regulated cravings may at least partially be explained through frontal brain oscillations. That is, the multiple mediation analysis signified a partial mediation effect of decision perspective on self-reported cravings through frontal alpha power. This denotes that in particular augmented activity in the alpha frequency range is associated with increased cravings of high-calorie foods and potentially unhealthy eating behavior. Additionally, irrespectively of behavioral ratings, we found that during delayed (vs. immediate) gratification of food rewards, i.e., in later-trials, the PSD in both the theta and alpha frequency spectra as well as FAA were increased.

This is in line with previous neuroimaging research using the ROC, which has shown increased BOLD activation in frontal regions associated with cognitive control, including the dmPFC, dlPFC, and vlPFC (Kober et al., 2010b). A hyperactivation of these regions might therefore denote cognitive overload (Matsuo et al., 2007). In fact, structural MRI studies have consistently reported reduced gray matter volume in these frontal regions (Horstmann et al., 2011; He et al., 2013) as well as lower structural connectivity between frontal and limbic structures associated with decision-making, reward, and interoceptive awareness (Gupta et al., 2015; Peng-Li et al., 2020c) in individuals with elevated impulsivity and poorer self-regulation abilities. An EEG study by (Meule et al., 2013) also found larger LPP amplitude (350–550 ms after onset)—an ERP component commonly linked to attention capture (Zorjan et al., 2020) and emotion regulation (Hajcak et al., 2010).

Likewise, empirical findings in consumer neuroscience, popularly referred to as neuromarketing, have utilized FA and FAA to objectively quantify consumer behaviors (Bazzani et al., 2020), such as willingness to pay (Ramsøy et al., 2018), hedonic food valuation (van Bochove et al., 2016), and attention biases (McGeown and Davis, 2018). This suggests that FAA cannot only be used as a measure of cognitive engagement but also as an emotional valence marker denoting affective and reward processes, including anticipatory pleasure and incentive salience (“wanting”). Although, one might expect that the FAA ought to be greater during now-trials due to closer reward proximity and delayed discounting, the manifestation of the opposite pattern can be reasoned through higher incentive salience and valuation of health benefits. That is, participants may have considered the future rewards of controlling their consumption of unhealthy foods in the presence. Nevertheless, this evidence, across different neuroimaging modalities and metrics, suggests increased cognitive demand and emotional engagement, especially when actively deliberating on long-term consequences (system 2) rather than simply evaluating immediate rewards in the present (system 1).

Importantly, these psychophysiological processes may be even more intensified during exteroceptive sensory inputs and distractions including ambient noise, as the increased theta activity in the later-trials was only occurring in the presence of loud (vs. soft) ambient noise. Correspondingly, alpha activity was also augmented during the loud noise condition, yet serving as a main (and not interaction) effect. As theta and alpha waves are arguably the power spectra mostly associated with cognitive/work load and attention (Klimesch, 1996; O’Keefe and Burgess, 1999; Stipacek et al., 2003; Antonenko et al., 2010; Brouwer et al., 2012; Wang et al., 2019), a combination of reflective system 2 thinking during prospective thinking and environmental auditory disturbances requires the most cognitive resources.

However, the power of the cerebral oscillations in the higher beta frequency spectra was not affected by decision perspective but solely augmented in the loud noise condition. Salisbury et al. (2002) similarly observed that background noise increased the latency of the P300 component, even while performance was unaffected. In an EEG review, Blume et al. (2019) have highlighted the elevated resting-state beta activity in fronto-central regions in individuals with obesity and binge-eating disorder. The authors argued that this increased beta activity may be the manifestation of the hyper-awareness of food cues and maladaptive eating behavior. Through deductive reasoning and in light of these collateral findings in combination with the results from the present study, it can be inferred that excessively loud noise indeed has neurophysiological impacts. This is measured by means of augmented beta activity, which in turn may provoke adverse effects on food-seeking behavior, even though we did not establish that link between beta activity (only alpha) and behavior (cravings) in the mediation analysis.

In addition, we found that the probability for EDA peak detection was also higher during the exposure to loud noise, indicating elevated arousal state (Salimpoor et al., 2011; Kantono et al., 2019). Louder noise may lead to a more stressful mindset that in turn diminishes the cognitive resources requisite for processing and making more rational and healthy decisions (Caviola et al., 2021). In contrast, when consumers are not interrupted by loud restaurant noises, they are in a more relaxed psychological state, which places them in a better position of restraining and managing their irrational and unhealthy food choices (Peng-Li et al., 2021). In fact, fast tempo and high volume of sound, both of which elevate physiological arousal (Liu et al., 2018; Biswas et al., 2019), have been reported to reduce one’s cognitive abilities, such as decision accuracy (Day et al., 2009), task performance (Nagar and Pandey, 1987), and creative thinking (Mehta et al., 2012).

Altogether, the findings are partly in line with our hypothesis that both noise level and decision perspective would influence subjective food cravings and objective measures, including EDA and EEG. However, we did not observe that the manipulations of both noise level and decision perspective had an impact on all measures. Indeed, alpha activity was affected by both loud noise and prospective thinking and could even predict food cravings based on the mediation analysis. Theta activity was influenced by the interaction of these, i.e., only loud noise and later decision perspective. Yet, beta activity and EDA peak probability were solely determined by noise level, while FAA and food cravings were influenced by decision perspective only. Hence, it can be inferred that louder noise and prospective thinking strategy can at least to some degree elevate neurophysiological constructs of emotional arousal and motivation as well as cognitive load, but will not necessarily help consumers regulate and manage their ultimate subjective food cravings.

### Managerial Implications

Due to the interdisciplinary nature and methodological novelty of our study, the results have several translational implications both clinically and commercially. First, we have demonstrated that food cravings could be restrained effectively merely via a single cognitive strategy involving deliberately devaluing the immediate rewards and delaying one’s gratification for future and long-term health benefits. Thus, we build on the previous literature that has incorporated cognitive strategies to highlight the use of interoceptive regulation and managerial decision-making in food (Kober et al., 2010b; Meule et al., 2013; Boswell et al., 2018) and other substance (Kober et al., 2010a; Naqvi et al., 2015; Suzuki et al., 2020) cravings, which collectively reinforces the theoretical foundation for practically implementing these measures in clinical contexts to help individuals who exhibit maladaptive eating behaviors.

Secondly, the identified underlying neurophysiological mechanisms by which top-down self-regulation alleviates cravings, are essential for understanding people’s subconscious and at times suboptimal eating behaviors. In addition, by applying exteroceptive auditory manipulations that analogously affect these fronto-cortical brain oscillations, we emphasize the importance of the power of a well-engineered acoustic environment. Hence, managers and other practitioners, who are at least partly responsible for the consumer, could try to establish eating atmospheres that reinforce healthier eating behavior by reducing stress, arousal and mental load (Doucé and Adams, 2020). Especially, in the times of COVID-19, in which several patients have suffered from anosmia (i.e., loss of smell) and/or ageusia (i.e., loss of taste), focusing on other attributes of the food, such as the texture, could help regaining the hedonic eating experience (Høier et al., 2021). Broaden out, one could also imagine that auditory cues, both intrinsic (i.e., the inherent sound of the food) and extrinsic/contextual (e.g., background music), might sensorily compensate for the loss of olfactory and gustatory perception.

Finally, with the current study being a cross-over between sensory and consumer science and cognitive neuroscience, the framework of the experiment in itself advocates the relevance of robust multidisciplinary research in decision sciences. Particularly, there has been increasing employment of neuroimaging procedures and biometric measurements in (food) market research (Knutson et al., 2007; Plassmann et al., 2008; Clement et al., 2013; Motoki and Suzuki, 2020), and neuromarketing and neuroeconomics have received considerable attention in both the scientific community and the media (Platt and Huettel, 2008; Ariely and Berns, 2010; Plassmann et al., 2012; Zhang et al., 2019). Thus, with the implementation of both EEG and EDA measurements, the study is of commercial and managerial interest. These tools can offer objective quantitative insights beyond traditional subjective and explicit methods that may be constrained by introspection and verbalization. From an industrial management perspective, consistent utilization of such multimodal methods might enable valid forecasting about consumers’ intentions, behaviors, and ultimately purchases. At the same time, it would at least to some degree increase reproducibility and circumvent the consequences of the replication crisis (Chives, 2019). Yet, to optimally exploit this attention and potential, while preventing it from becoming a mere marketing gimmick, academics in the respective fields should exploit their experience and ask relevant questions that can in fact provide useful inputs to marketers and managers in addition to conventional marketing research.

### Limitations

Despite these abovementioned implications, our study involves several limitations. First, it should be noted that the physiological signal analyses were based on rather conservative pre-processing procedures due to the employment of the integrated R algorithm of iMotions. This implicates inflexible parameter adjustments during data pre-processing of EEG and EDA. The EEG signal was referenced to a single mastoid instead of e.g., two mastoids or an average reference, but lateralized metrics, such as FAA can be prone to confounds (Lei and Liao, 2017). Analogously, we could not carry out scrutinized eye-blink detection, manual removal of single trials or events, nor independent component analysis (ICA), but only rely on the simple automated algorithm. Notably, according to a methodological review by Allen et al. (2004), for some spectral computations (e.g., FAA), artifact thresholding alone might be as adequate as using other manual accessorial procedures, such as electrooculography (EOG) and electromyography (EMG).

Secondly, due to the nature of our controlled experimental setup, our findings cannot necessarily be directly generalized to naturalistic food choice settings (Andrade, 2018) in which multiple other external factors (including price, labeling, and social factors) may affect the consumers’ emotional states, cognitive processing, and behaviors (Sørensen et al., 2013; Spence et al., 2014; Petit et al., 2015). Besides, albeit food cravings are strong predictors of eating behavior and food choice (Boswell et al., 2018; Chen et al., 2018; Sun and Kober, 2020), we cannot assure that these independent results encompass ecological validity and are applicable in a real-life managerial decision context.

Thirdly, we did not incorporate any neutral/silent condition, which could have strengthened the comparability within the study, as done in some previous food, sound, and decision research (Alamir et al., 2020; Peng-Li et al., 2020a). However, the longer design could have been time-consuming and fatiguing for the participants. Besides, one could argue that the soft noise condition would serve as a control condition since complete silence is highly unlikely in a normal eating situation.

Finally, we simply confined our EEG analyses to the frontal part of the brain through theta, alpha, and beta activity based on our conceptual framework. While, several studies have investigated the oscillatory power in other or smaller ROIs (Tashiro et al., 2019; Biehl et al., 2020) as well as other frequency bands (i.e., delta and gamma; Colrain et al., 2009; Dimigen et al., 2009) during mental operations, we chose not to, as the analyses would be unreasonably extensive and outside the scope of our framework.

## Conclusion

To conclude, the present study has underlined the combined effects of cognitive regulation and ambient restaurant noise on food cravings through EDA peak probability as well as fronto-cortical brain oscillations as quantitative measures of emotional arousal, motivation, and cognitive load. More broadly, we have highlighted the prospect of and need for considering both interoceptive states and exteroceptive cues, while employing different physiological measurements to more holistically, objectively, and optimally study food-related decision-making that can provoke an actual societal and managerial impact. This is not solely confined to the field of sensory and consumer neuroscience, but for any decision sciences, this seems applicable and highly pertinent.

## Data Availability Statement

The raw data supporting the conclusions of this article will be made available by the authors, without undue reservation.

## Ethics Statement

The studies involving human participants were reviewed and approved by the Aarhus University Ethics Committee (approval number: 2020-0184772). The patients/participants provided their written informed consent to participate in this study.

## Author Contributions

DP-L: conceptualization, methodology, formal analysis, investigation, resources, data curation, project administration, writing—original draft, writing—review and editing, and visualization. PA: conceptualization and writing—review and editing. CC: investigation and writing—review and editing. RC: writing—review and editing and supervision. DB and QW: conceptualization, writing—review and editing, and supervision. All authors contributed to the article and approved the submitted version.

## Conflict of Interest

The authors declare that the research was conducted in the absence of any commercial or financial relationships that could be construed as a potential conflict of interest.

## Publisher’s Note

All claims expressed in this article are solely those of the authors and do not necessarily represent those of their affiliated organizations, or those of the publisher, the editors and the reviewers. Any product that may be evaluated in this article, or claim that may be made by its manufacturer, is not guaranteed or endorsed by the publisher.

## Footnotes

1
https://aucobe.sona-systems.com/default.aspx?logout=Y

2
https://imotions.com

3
http://elastix.isi.uu.nl/

4
https://freesound.org
